# Preparation, formation mechanism, photocatalytic, cytotoxicity and antioxidant activity of sodium niobate nanocubes

**DOI:** 10.1371/journal.pone.0204061

**Published:** 2018-09-14

**Authors:** Muhammad Nawaz, Sarah Ameen Almofty, Faiza Qureshi

**Affiliations:** 1 Department of Nano-Medicine Research, Institute for Research and Medical Consultations (IRMC), Imam Abdulrahman Bin Faisal University, Dammam, Saudi Arabia; 2 Department of Stem Cell Research, Institute for Research and Medical Consultations (IRMC), Imam Abdulrahman Bin Faisal University, Dammam, Saudi Arabia; 3 Deanship of Scientific Research, Imam Abdulrahman Bin Faisal University, Dammam, Saudi Arabia; Institute of Materials Science, GERMANY

## Abstract

A hydrothermal method was employed to prepare the sodium niobate (NaNbO_3_) nanocubes. We executed time dependent experiments to illustrate the formation mechanism of sodium niobate nanocubes. It was observed that the morphology of NaNbO_3_ nanocubes was dependent on the reaction time and 12hr reaction time was found to be suitable. Morphology, composition, structure and optical properties of sodium niobate nanocubes were evaluated by scanning electron microscope, X-ray energy-dispersive spectrometer, X-ray diffraction and UV-visible diffuse reflectance spectrometer. The photocatalytic activity of sodium niobate was studied for photocatalytic hydrogen production. It was anticipated that the sodium niobate (NaNbO_3_) cubes exhibited good photocatalytic activity under UV light irradiation using lactic acid as sacrificial agent. The cytotoxicity activity of sodium niobate nanocubes was studied as well at different concentrations (5 mg/mL, 3 mg/mL, 1 mg/mL, and 0.25 mg/mL) against human colon colorectal carcinoma cell line (HCT116) by MTT assay and EC_50_ was found to be 1.9 mg/mL. Sodium niobate proved to be a good DPPH free radical scavenging material, tested at different concentrations. It was noticed that peak intensity at 517 nm was decreased after 30 minute incubation, further supporting the antioxidant activity. This study will be useful for design and engineering of materials that can be used in biomedical applications and in photocatalysis.

## Introduction

Excess consumption of fossil fuel and increased pollution has make scientists actively looking for solutions in terms of energy and environmental issues. Hydrogen is as an eco-friendly fuel and an alternative source of energy. Currently, hydrogen is mostly used as energy source via fossil fuels and electrolysis of water, the use of latter, however is economically not feasible as the process consumes high energy. Solar energy is the alternatively favored renewable energy source that can satisfy the global energy requirements. In order to resolve energy crisis and environmental problems, photocatalytic hydrogen evolution using photocatalysts of semiconductors such as oxides, sulfides and nitrides has been considered as an attractive and clean way, low-cost and eco-friendly production of hydrogen [1–5].

The engineering and design of nanomaterials with controlled morphology has been getting more attention and significance owing to their unique physical and chemical properties. The photocatalytic properties of nanomaterials are vastly dependent on shape, size, crystal facets, size distribution and phases [6–10] which makes it much more challenging for the researchers to prepare the perfectly engineered materials that can be used as biomaterial, photocatalyst, catalyst support and adsorbent [11–18].

Sodium niobate (NaNbO_3_) is a fascinating material with pervoskite structure at a low cost. It has high crystallinity and good chemical stability with low environmental impact [19–21], attracts scientists due to its ionic conductivity, photorefractive properties, nonlinear optics and photocatalytic properties [22–25]. Different morphologies of nano NaNbO_3_, such as nanoplates, nanocubes and nanowires have been studied for photocatalytic activities with the conclusion that photocatalytic activity improved with controlled morphology of NaNbO_3_ [26–29]. Current research indicates that sodium niobate is an efficient photocatalyst for hydrogen production [30], photocatalytic decomposition of organic contaminants [25] and CO_2_ reduction [30–32].

Several methods had been reported for the preparation of sodium niobate (NaNbO_3_), however, most of them involved complicated polymerized complex methods [32, 33], and therefore, the need to develop simple and economic method for the preparation of sodium niobate (NaNbO_3_) was felt. As we discussed above, to resolve the energy crisis there is need to prepare the low-cost and eco-friendly photocatalyst for hydrogen production. This study involved the preparation of sodium niobate (NaNbO_3_) by hydrothermal method and its application as photocatalyst and biomaterial. Furthermore, the formation mechanism and cytotoxicity of sodium niobate has never been explored and studied so far. This study is useful to provide the details of formation mechanism of NaNbO_3_ and to study the cytotoxicity, antioxidant and photocatalytic activity.

## Materials and methods

### Chemical and materials

All chemicals, kits and reagents were purchased from commercial sources and used as it.

### Preparation of sodium niobate cubes

A mixture of niobium pentoxide (Nb_2_O_5_, 0.265g) and sodium hydroxide (0.6g), was stirred in deionized water at room temperature for certain period. The solution was then transferred into Teflon-lined autoclave and heated at 200°C for 12h. After cooling, Teflon-lined autoclave at room temperature; the white precipitates obtained were centrifuged and washed several times with deionized water and ethanol. Lastly, the product was dried at 60°C for 12h.

### Characterization

X-ray diffraction pattern of NaNbO_3_ was recorded on a Rigaku X-ray diffractometer using Cu Kα radiation. Morphology of the product was observed by field emission scanning electron microscope (FE-SEM) and elemental composition was determined by X-ray energy-dispersive (EDX). The UV-visible spectrum was obtained on Shimadzu UV-Vis spectrophotometer using BaSO_4_ as a reference.

### Photocatalytic activity

Photocatalytic activity of NaNbO_3_ was studied in a closed gas circulation system under a 300W xenon lamp. 50 mg of catalyst was added to aqueous lactic acid solution and dispersed ultrasonically. The solution was evacuated for 20–30 minutes to remove the dissolved gases before irradiation and then exposed to irradiation by Xe lamp. The hydrogen gas generated was determined *in situ* by a gas chromatogram (TECHCOMP, GC 7890-II) equipped with a TCD detector [8].

### Cell culture and cytotoxicity activity

Human colon colorectal carcinoma cell line (HCT116) was purchased from ATTC (American Type Culture Collection, USA) and maintained in DMEM medium. Cytotoxicity activity of NaNbO_3_ nanocubes were evaluated against HCT116 cells by MTT assay Vybrant^®^ MTT Cell Proliferation Kit (Thermo Fisher Scientific). HCT116 cells were seeded in 96-well plates at (10^4^ cells/well) in DMEM medium supplied with 10% fetal bovine serum and 1% penicillin-streptomycin. Different concentrations of NaNbO_3_ nanocubes (5 mg/mL, 3mg/mL, 1 mg/mL, and 0.25 mg/mL) were added to the wells. Cells were maintained in humidified atmosphere with 5% CO_2_ at 37°C and incubated for 24 h. After incubation, culture medium was removed and fresh medium was added with 10 μL of MTT solution, cells were further incubated for 4h at 37°C. Medium was removed after incubation and sterile DMSO (100 μL) was added to solubilize the formazan blue crystals. SYNERGY Neo2 multi-mode microplate reader (Biotek) was employed to record the absorbance at 570 nm. Cell viability was then calculated using following formula:

Cellviability(%)=absorbanceofsample/absorbanceofcontrol*100

### Antioxidant activity

The antioxidant activity of NaNbO_3_ was studied using DPPH as free radicals source. To assess the antioxidant activity of NaNbO_3_, different concentration such as 5 mg/mL, 3 mg/mL and 1mg/mL were prepared. 1 mL of DPPH solution (0.1 mM in methanol) was mixed with 3 mL of NaNbO_3_ different concentrations and incubated in dark for 30 minutes. Mixture was centrifuged and supernatant was analyzed at 517 nm against blank using UV-visible spectrophotometer. The % inhibition of DPPH scavenging activity was computed as:

$$\%\;\text{DPPH}\;\text{radical}\;\text{scavenging}\;\text{activity}=\left({\text{Abs}}_{\text{blank}}-{\text{Abs}}_{\text{sample}}/{\text{Abs}}_{\text{blank}}\right)*100$$

Where Abs _blank_ is the absorbance of blank and Abs _sample_ absorbance of sample.

## Results & discussion

### Characterization

**Fig 1:**
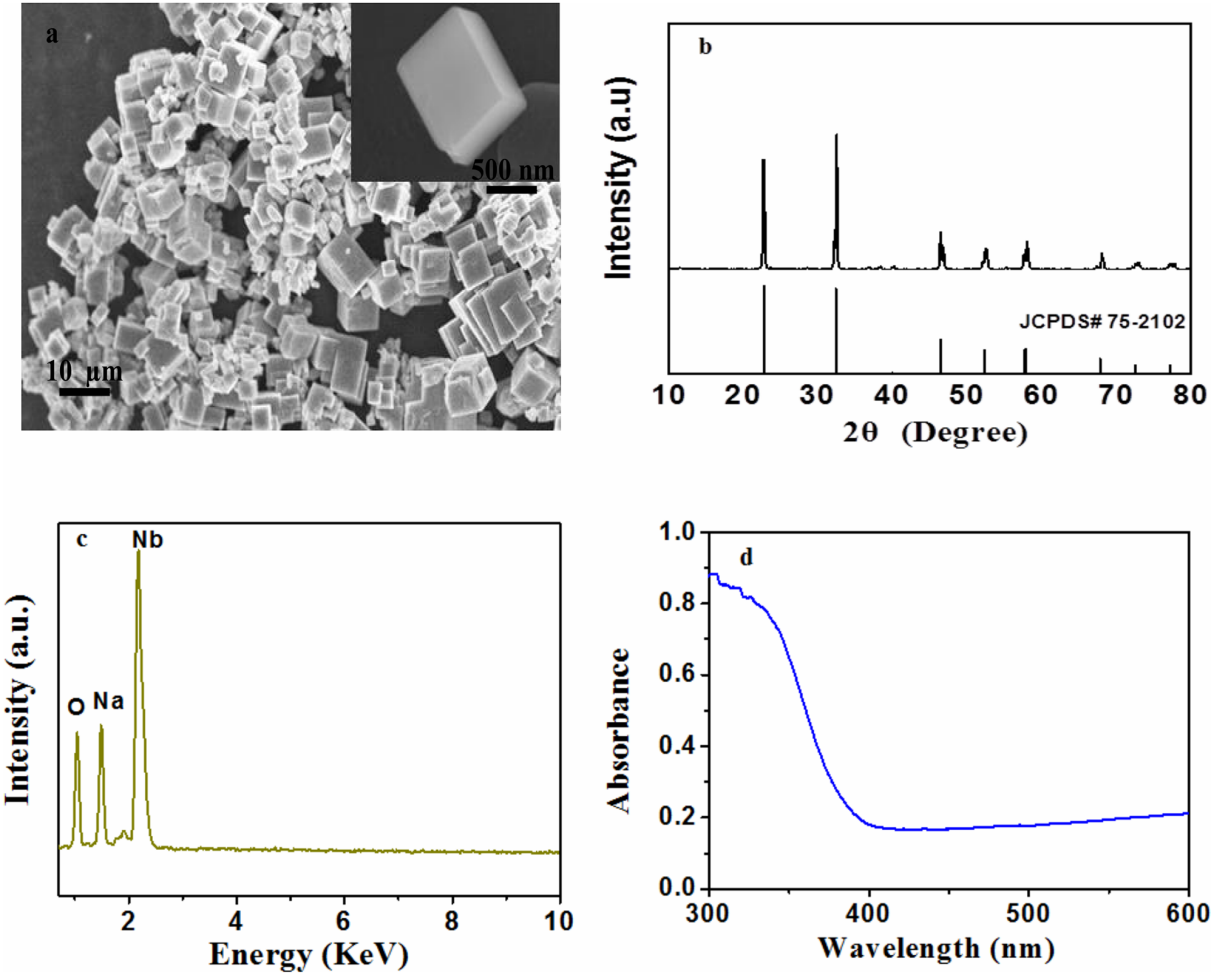
Characterization data of NaNbO_3_. (a) FESEM images of NaNbO_3_. (b) XRD pattern of NaNbO_3_. (c) EDX pattern of NaNbO_3_. (d) DR-UV-visible spectrum of NaNbO_3_.

NaNbO_3_ nanocubes were prepared by hydrothermal method and morphology of the product was observed by SEM. Fig 1a shows representative images of NaNbO_3_ nanocubes at different magnifications. It is clear from SEM images that the product has nanocubes morphology with 500 nm in size.

The crystal structure of NaNbO_3_ was probed by X-ray diffraction. Fig 1b shows the XRD pattern of NaNbO_3_ prepared at 200°C for 12 h. The sharp and strong diffraction peaks suggest a nanocrystalline structure. All the diffraction peaks can be indexed as cubic phase of NaNbO_3_ (JCPDS card no. 75–2102). No impurities can be detected in this pattern, which implies cubic phase of NaNbO_3_ can be obtained under the described experimental conditions.

Energy-dispersive X-ray spectroscopy (EDX) was performed to confirm the composition of the as-prepared product. We selected different surface as collection areas and found the same results. Fig 1c shows energy-dispersive X-ray spectroscopy (EDX) spectra of NaNbO_3_; confirming the presence of Na, Nb and O in the product and also shows there is no impurity in the product. A UV-visible diffuse reflectance (DRS) spectrum of NaNbO_3_ is revealed in Fig 1d. The diffuse reflectance spectra of the product demonstrated an absorption edge at 386 nm in the UV region, corresponding to the band gap energy of 3.21 eV.

### Formation mechanism

**Fig 2:**
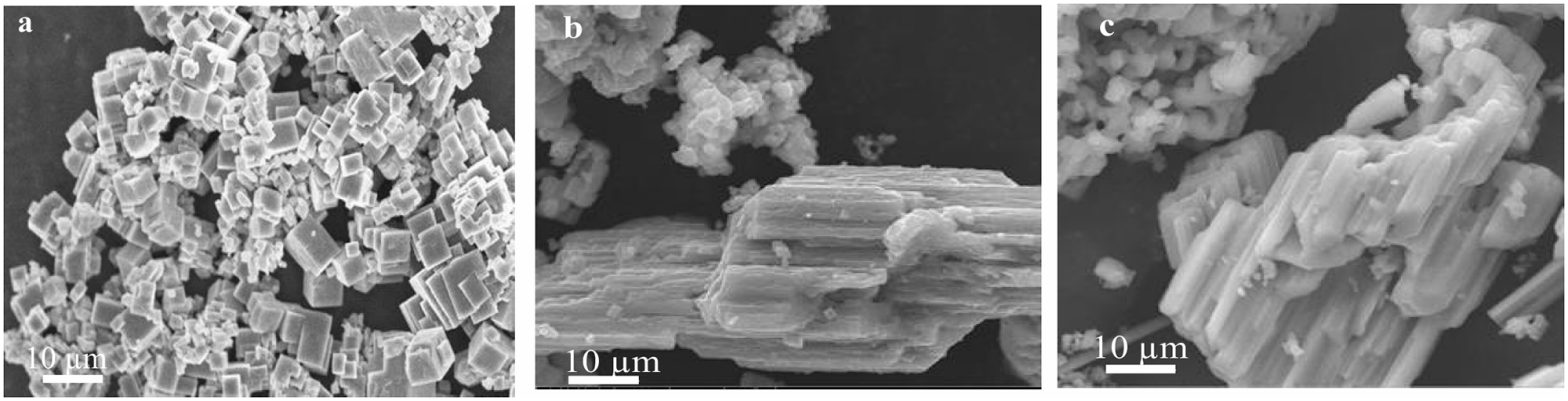
Effect of solvents on the morphology and size of NaNbO_3_. (a) Water. (b) Methanol. (c) Ethanol.

**Fig 3:**
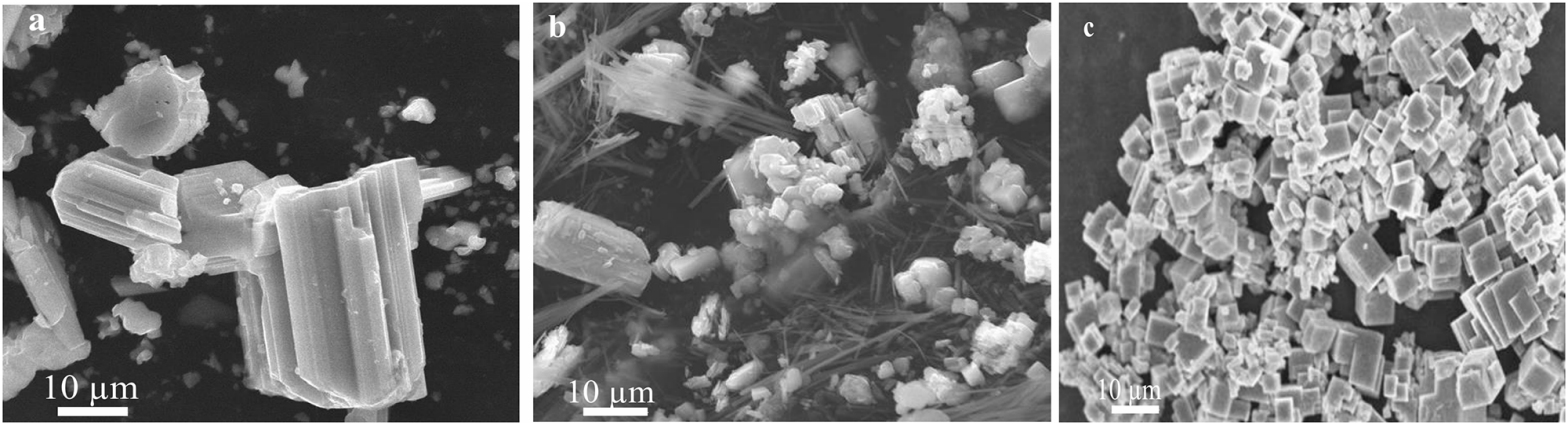
Effect of temperature on the morphology and size of NaNbO_3_. (a) 150°C. (b) 180°C. (c) 200°C.

**Fig 4:**
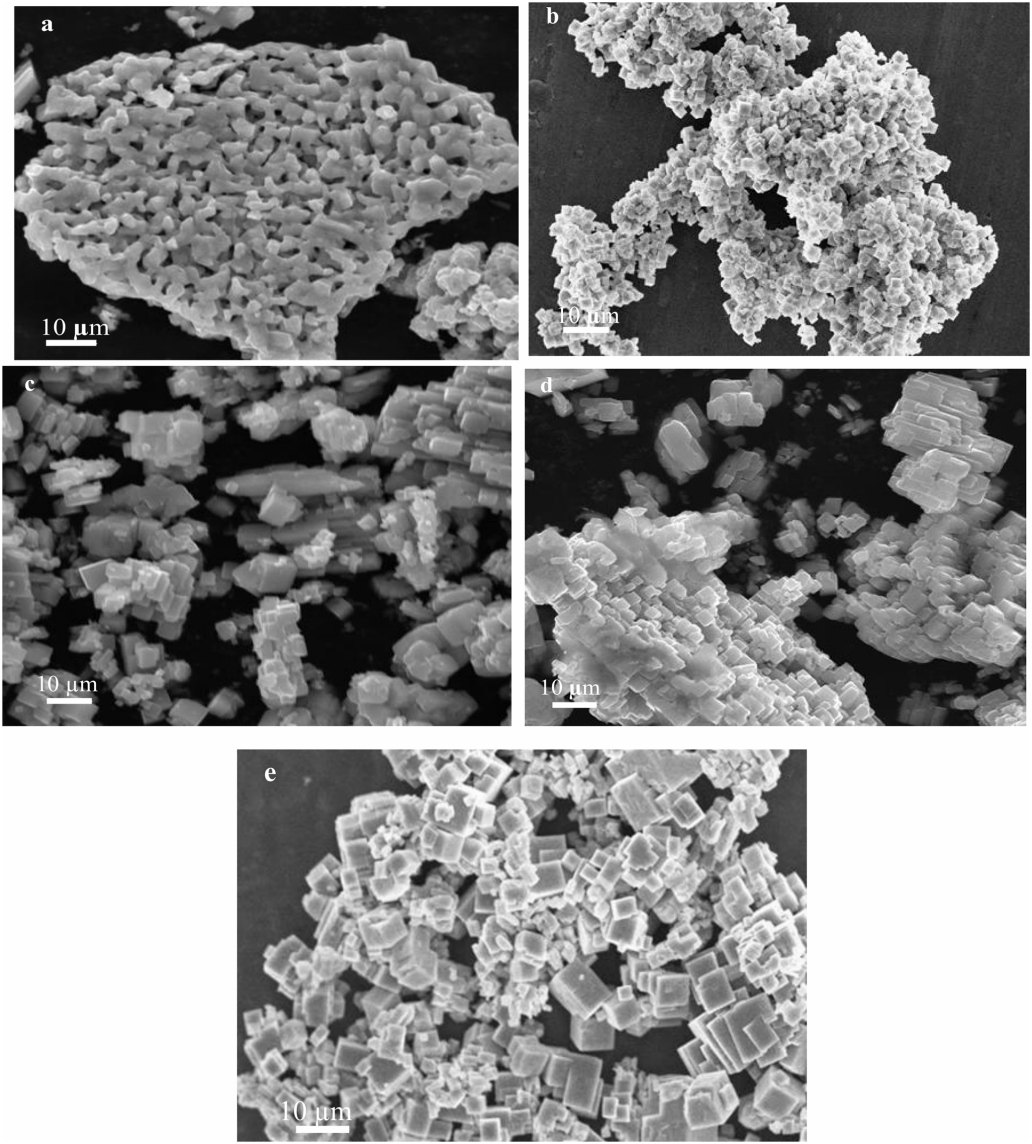
FESEM images of NaNbO_3_ at different times. (a) 30 min. (b) 1h. (c) 3h. (d) 6h. (e) 12h at 200°C.

In order to check the effect of different solvents on the morphology and size of NaNbO_3_, experiments were conducted in water, methanol and ethanol at 200°C. SEM results obtained in different solvents are shown in Fig 2a–2c. As we can see in the Fig 2a, when reaction was conducted in the water, the product was nanocubes. On the other hand, when reaction was performed in methanol and ethanol, nanocubes were not formed (Fig 2b and 2c). Hence water was selected as a reaction medium for the preparation of NaNbO_3_ nanocubes. To optimize the reaction temperature and to see the effect of temperature on the morphology and size of NaNbO_3_ nanocubes, we performed the reaction at different temperatures (150°C, 180°C and 200°C). SEM images of the products obtained at 150°C, 180°C and 200°C are shown in the Fig 3a–3c. It is clear from SEM results, the product obtained at 150°C has big block like morphology with aggregates (Fig 3a). On the other hand, when reaction was performed at 180°C, nanorods and aggregated nanocubes were formed (Fig 3b). Furthermore, the product obtained at 200°C was consisted of nanocubes (Fig 3c). These results suggested that reaction temperature 200°C is optimal to get NaNbO_3_ nanocubes. To further understand the formation mechanism of NaNbO_3_ nanocubes at 200°C, we conducted reactions at different times. As it is evident from Fig 4a, the product obtained after 30 minute of reaction comprises of spheres. After 1h, nanocubes are formed with the co-existence of small particles (Fig 4b). The SEM image in Fig 4c indicates that nanocubes are formed as the reaction proceeded for 3 h. Fig 4d is SEM image of nanocubes formed after 6 h reaction time with some aggregates. Finally, well-developed nanocubes (having size from 100–500 nm) were observed upon increasing further reaction time to 12h (Fig 4e).

**Fig 5:**
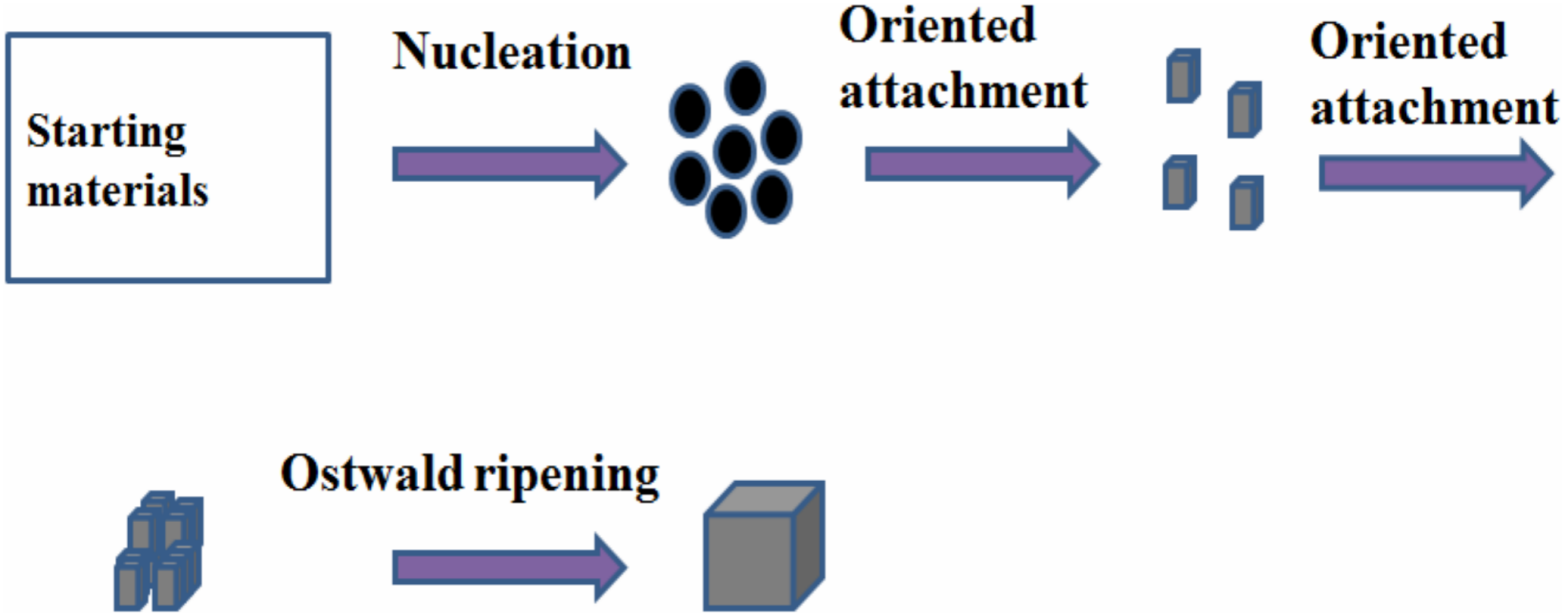
Stepwise illustration for the formation mechanism of NaNbO_3_ nanocubes.

Based on the above discussion, it can be deduced that oriented attachment and Ostwald ripening are involved for the formation of NaNbO_3_ nanocubes. Fig 5 displays proposed formation mechanism for NaNbO_3_ nanocubes. In the course of reaction, NaOH reacts with Nb_2_O_5_ and it results in the formation of primary nanocrystals. The primary nanocrystals undergo oriented attachment to form small cubes. These primary nanocubes undergo mutual orientation and self-assembled to form nanocubes (Fig 5). When reaction was preceded further, reaction rate further decreased and at this stage Ostwald ripening became more dominant. Due to higher surface energy, small particles slowly dissolved to form nanocubes [34]. Stepwise formation mechanism is illustrated in Fig 5.

### Photocatalytic activity

**Fig 6:**
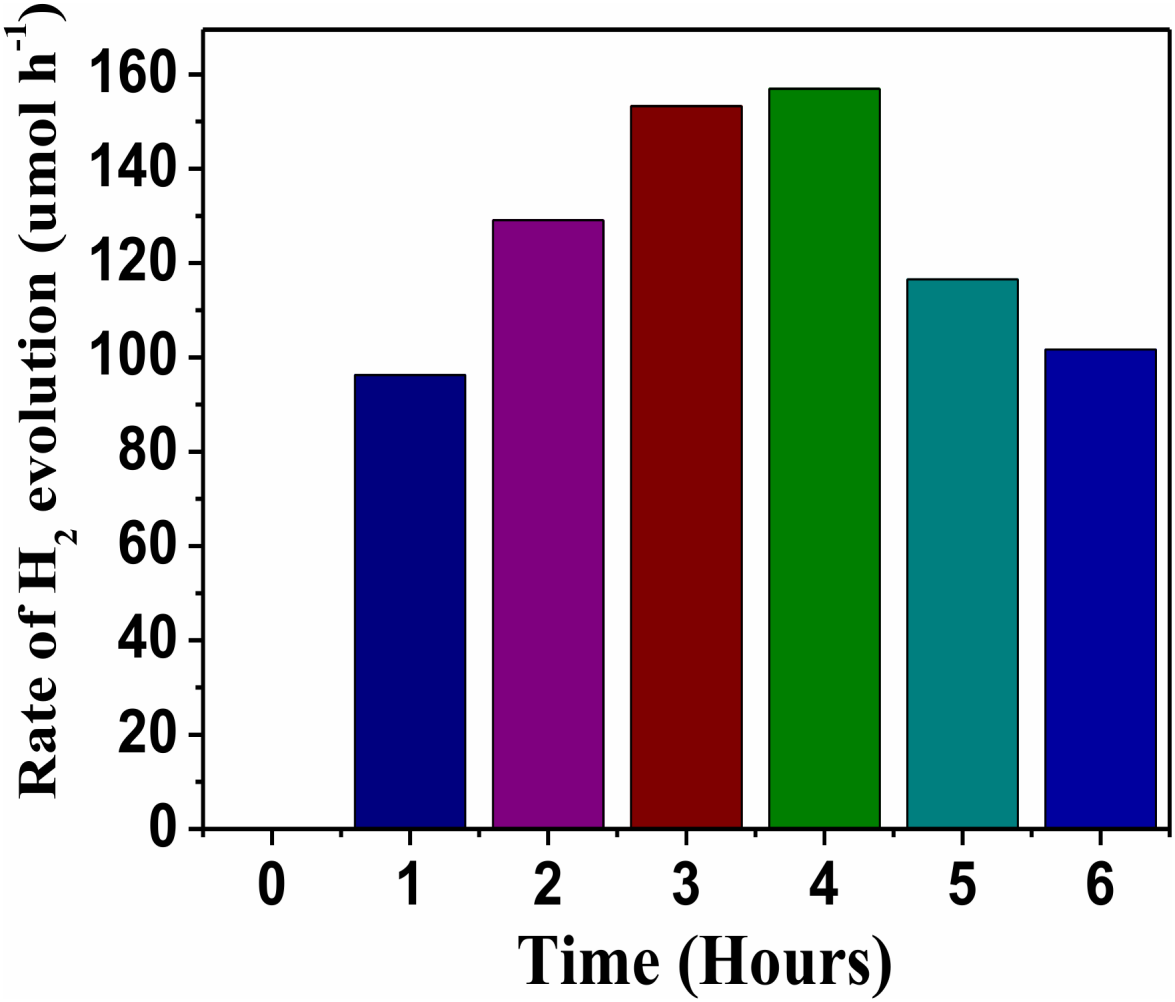
H_2_ evolution rate by NaNbO_3_ for 6 h irradiation.

**Fig 7:**
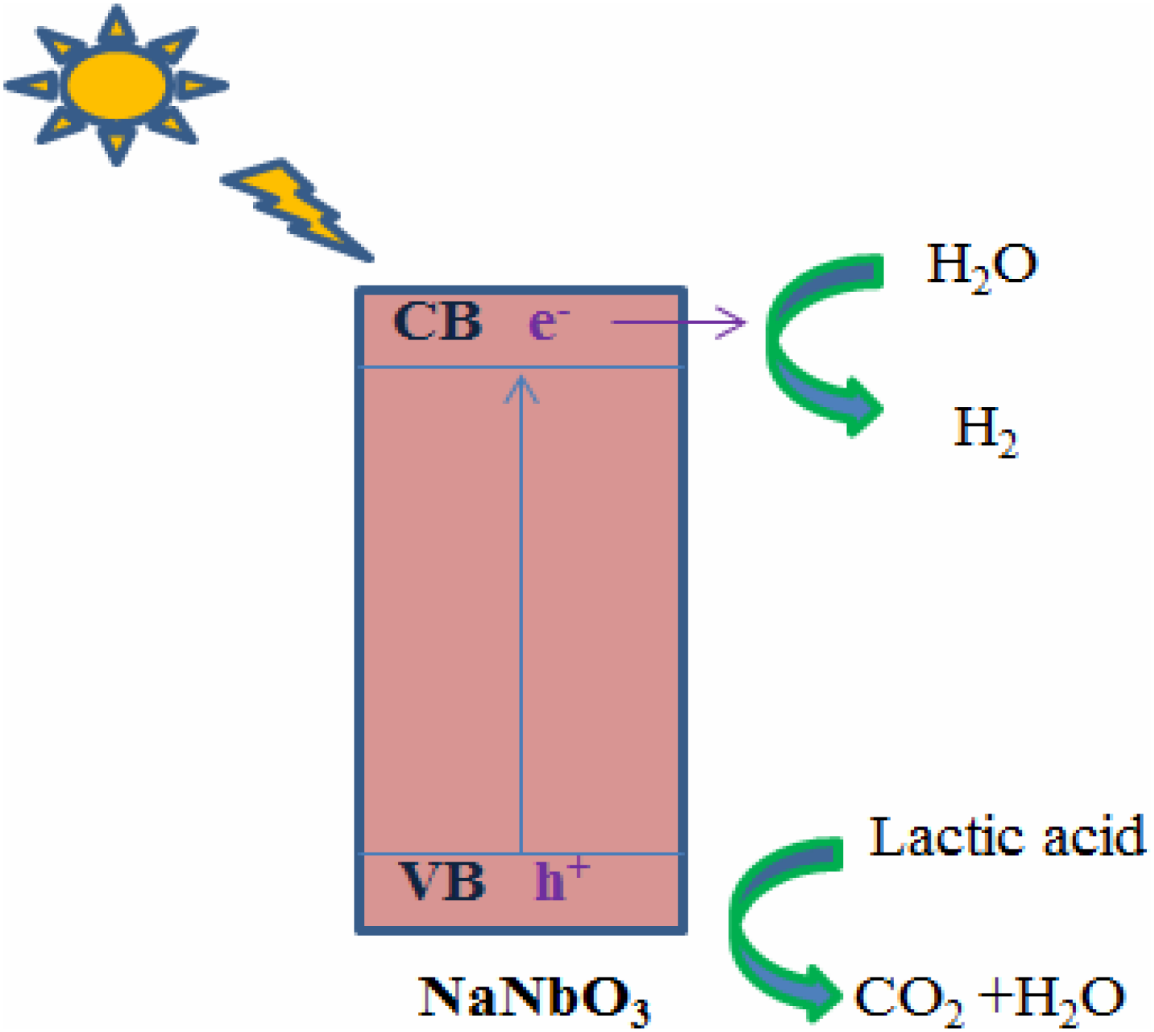
Proposed mechanism for the photocatalytic hydrogen production of NaNbO_3_ under UV light irradiation using lactic acid as sacrificial agent.

The photocatalytic activity of synthesized NaNbO_3_ nanocubes for hydrogen production was evaluated under UV light and expressed in terms of hydrogen evolution rate (Fig 6). As it can be seen in Fig 6, the photocatalytic activity of NaNbO_3_ started increasing with passage of time. However, after certain time the photocatalytic activity was dramatically decreased. It could be possible that with the lapse of time the concentration of sacrificial reagent was decreased which result in the lower rate of hydrogen production. The photocatalytic activity of NaNbO_3_ could be ascribed to its light harvesting ability and can accelerate the transportation of photo-generated electron hole pairs, making the photocatalytic process more efficient [9]. Furthermore, good crystallinity of NaNbO_3_ also reduces the chance of electron hole recombination and enhances the photocatalytic activity. Proposed mechanism for the photocatalytic hydrogen production of NaNbO_3_ under UV light irradiation is illustrated in Fig 7.

### Cytotoxicity activity

**Fig 8:**
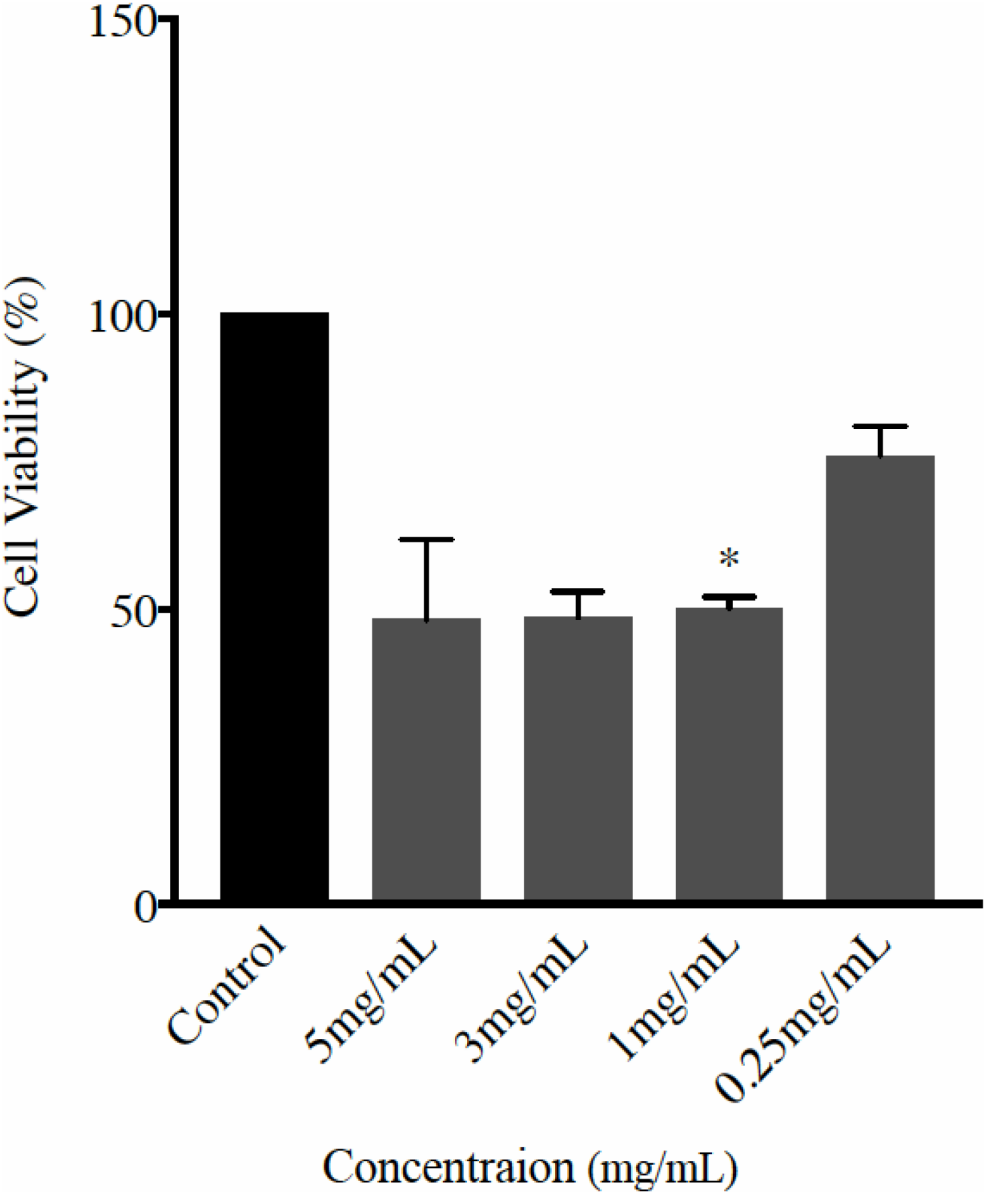
Percentage of cell viability using MTT assay. HCT116 cells were treated with different concentrations of NaNbO_3_ nanocubes (5 mg/mL, 3 mg/mL, 1 mg/mL and 0.25 mg/mL) and MTT assay was performed 24h post incubation to measure the cell viability. The statistical analysis was performed using oneway ANOVA- Dunnett’s test, whereas * = p<0.05.

The cytotoxicity activity of NaNbO_3_ nanocubes was studied against human colon colorectal carcinoma cell line (HCT116) at different concentrations such as 5 mg/mL, 3 mg/mL and 1 mg/mL and 0.25 mg/mL. The cytotoxicity activity of NaNbO_3_ nanocubes was observed from 1 mg/mL concentration and at the higher concentrations, whereas no detectable effect at the lowest concentration (0.25 mg/mL) was observed. The effective concentration (EC_50_) of NaNbO_3_ was calculated using the equation generated from exponential chart of the cell viability percentage against concentrations. EC50 was found 1.9 mg/mL. The cell viability % of the NaNbO_3_ nanocubes treated cells at different concentration is shown in Fig 8.

### Antioxidant activity

**Fig 9:**
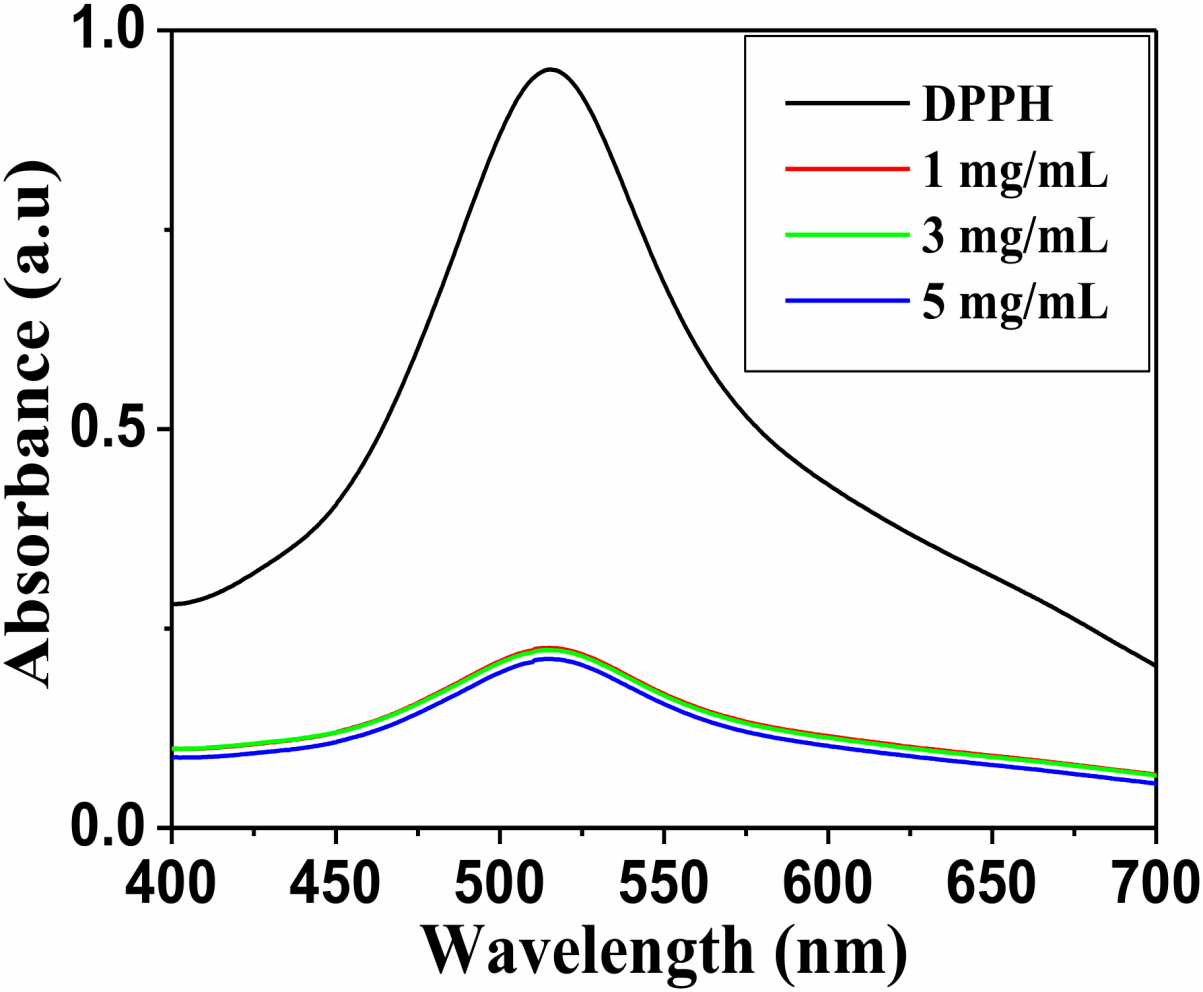
UV-visible spectra indicating antioxidant activity (Reduction in peak intensity) of NaNbO_3_ nanocubes at different concentrations.

We considered different concentration of NaNbO_3_ nanocubes to study the antioxidant activity. During the experiment, a change in color of DPPH solution from violet to pale yellow was noticed in the presence of NaNbO_3_ nanocubes. Furthermore, intensity of peak at 517 was decreased after 30 minute (Fig 9), indicated the free radical scavenging activity of NaNbO_3_ nanocubes. The free radical scavenging activity of NaNbO_3_ was perceived between 71.33 to 72.79% at different concentrations. The decline in intensity of peak at 517 nm could be attributed due to the transfer of electron density situated at oxygen to the odd electron found at nitrogen atom in DPPH.

The results of this study were promising; sodium niobate (NaNbO_3_) with controlled morphology (nanocubes) was prepared at 200°C. NaNbO_3_ revealed good photocatalytic activity for hydrogen production by splitting water indicating that it can be used as photocatalyst. The effective concentration (EC_50_) of NaNbO_3_ nanocubes against human colon colorectal carcinoma cell line (HCT116) was observed 1.9 mg/mL. NaNbO_3_ also demonstrated good antioxidant activity at different concentrations. Biological results indicate that NaNbO_3_ can be used as biomaterial.

## Conclusion

NaNbO_3_ nanocubes were successfully produced via facile hydrothermal method. Time dependent study was conducted to deduce the formation mechanism of NaNbO_3_ nanocubes. Results revealed development of nuclei that transitioned to segregated nanocubes as the reaction time progressed. Band gap of NaNbO_3_ was observed 3.21 eV and it exhibited good photocatalytic activity for hydrogen production under UV light irradiation. EC_50_ of NaNbO_3_ nanocubes against human colon colorectal carcinoma cell line (HCT116) was found to be 1.9 mg/mL. The antioxidant activity was found to be significant too and coupled with anticancer potential can be a good candidate as an engineered biomaterial. This work provides insights for the preparation of NaNbO_3_ nanocubes with well-regulated morphologies, serving as promising photocatalyst.
